# Supplementary material for: Antimicrobial Agent against Methicillin-Resistant Staphylococcus aureus Biofilm Monitored Using Raman Spectroscopy
Source: Pharmaceutics. 2023 Jul 12;15(7):1937. doi: 10.3390/pharmaceutics15071937 (PMC10384418; doi:10.3390/pharmaceutics15071937)
Supplement: Supplementary file 1 [file pharmaceutics-15-01937-s001.zip › pharmaceutics-2491480-supplementary.pdf]

Supplementary Material

Antimicrobial Agent against Methicillin-Resistant *Staphylococcus aureus* Biofilm Monitored Using Raman Spectroscopy

Jina Kim and Young-Won Chin *

*** Correspondence**: ywchin@snu.ac.kr; Tel.: +82-2-880-7859

1 Supplementary Figures

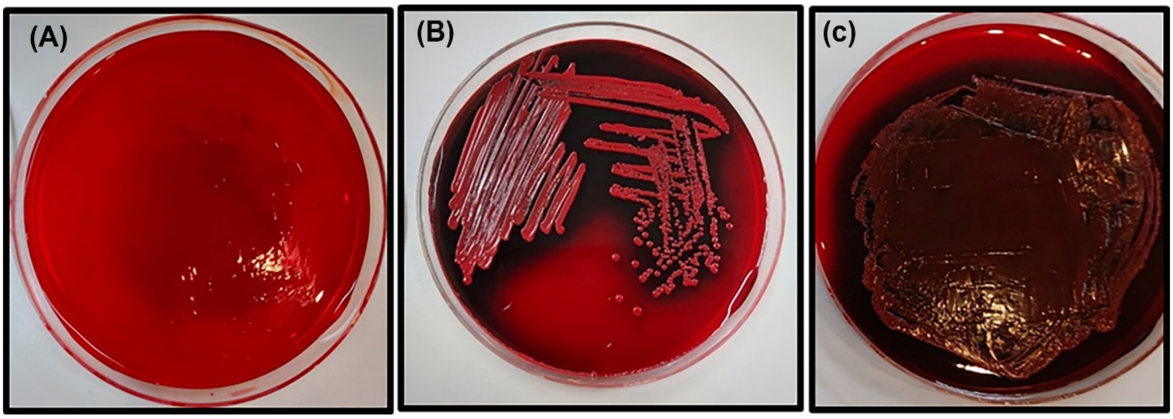

Supplementary Figure S1. (A–C) Phenotypic characterization of biofilm formation of MRSA strains on Congo red agar after 1, 24, and 48 h of incubation

In this research, PCA analysis gives a highly effective classification from initial planktonic cell to mature MRSA biofilm. Since planktonic bacterial cells are hard to distinguish from attached multicellular biofilm, PLS-DA was performed to find the correlation of the variables. The optimum number of latent variables is 8 in this study, and the method for estimating the latter number is k-fold cross validation. The explained variance in X and Y blocks, X is the wavelength of the Raman spectroscopy and Y variance biofilm phases. The class index matrix employed for training is class 1, and class 2. The class 1 includes from 1 to 53 sets that were classified as the planktonic bacteria in PCA analysis and the class 2 includes 117 sets of initial attached biofilm. The statistical indicators of class prediction for the test set of samples RMSEE(root mean square estimated error) = 0.073, RMSE(root mean square error) cv =
0.093

In SIMCA-P, the quality of PLS-DA model was described by the goodness-of-fit R2 (0 ≤ R 2 ≤ 1) and the predictive ability Q2 (0 ≤ Q 2 ≤ 1) values. An internal measure of model fit is provided by R2, and an internal measure of consistency to the cross-validation data is provided by Q2. In this study, PLS-DA models demonstrated high statistical values (R2 > 0.040 and Q2 ≥ 0.50), the difference between the goodness-of-fit and the predictive ability always remained below 0.3 (R2X(cum) − Q2 (cum) < 0.3) and the goodness of fit was never equal to one (R2X(cum) 9= 0.98). The cross-validation analysis of variance (CV-ANOVA) was used to validate the classification model, with a p-value < 0.05. Additionally, permutation tests were employed (500 permutations) in order to measure the effectiveness of the classification in a model by randomly permuting the original group attribution. All models were extracted at a confidence level of 95%. In this paper, the Receiver Operating Characteristics Curve (ROC) was drawn to validate the model. The area under the ROC curve equal to 1.

(A)

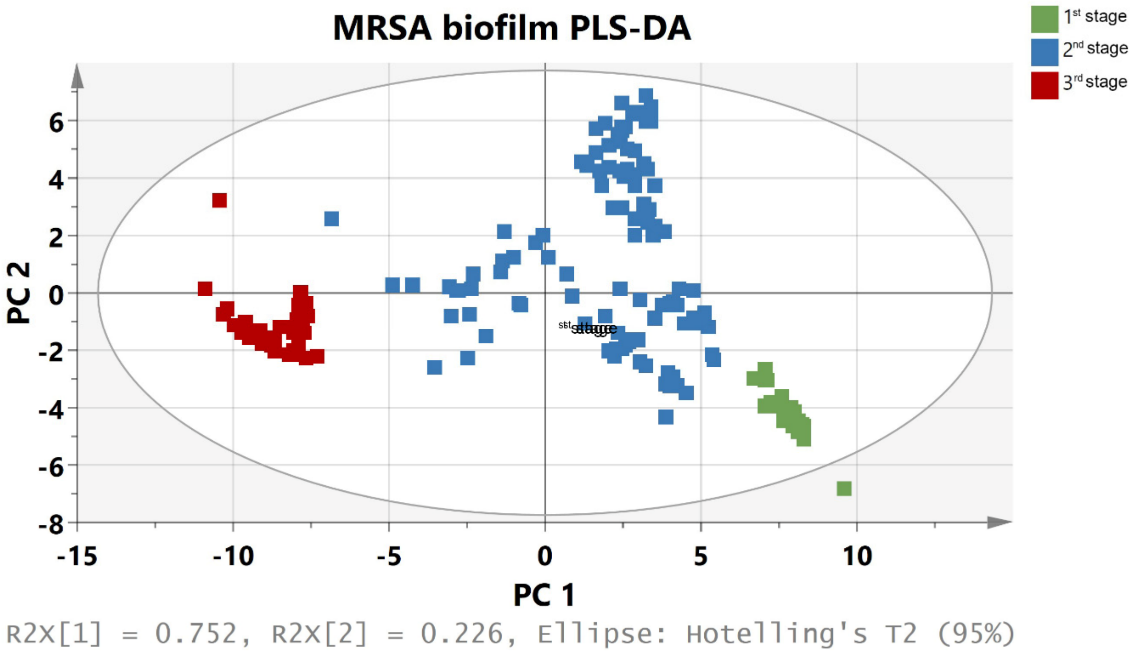

(B)

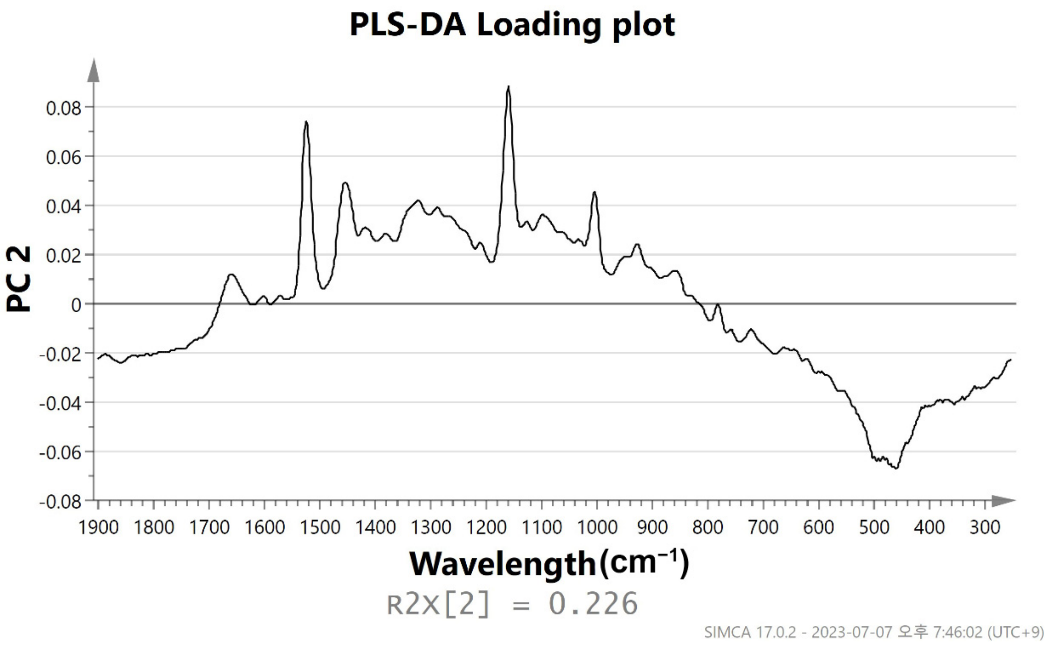

Supplementary Figure S2. (A) PLS-DA scores plot (biofilm groups 1, 2, and 3) with A = 1, N = 170, R2X(cum) = 0.99, R2Y(cum) = 0.96, Q2(cum) = 0.94 for Hotelling's T2 95% confidence level, p-value = 0 (B) Loading plot of component 2, R2X(2) = 0.226.

A

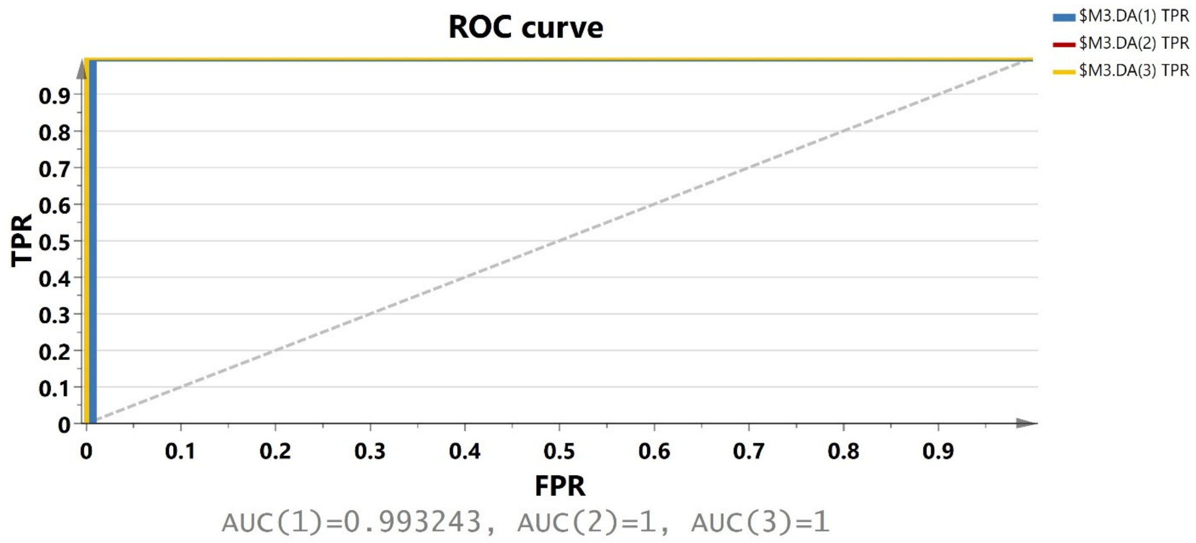

B

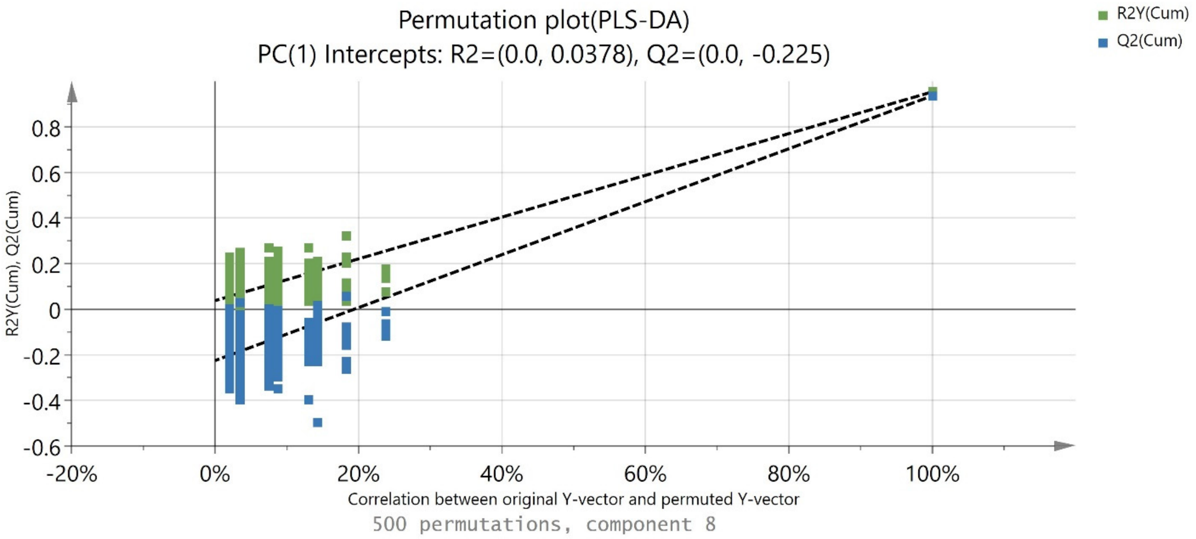

Supplementary Figure S3. (A) ROC curve AUC (Phase 1) = 0.99 AUC (Phase 2) = 1 AUC (Phase 3) = 1. (B) Permutation test for the PLS-DA model.

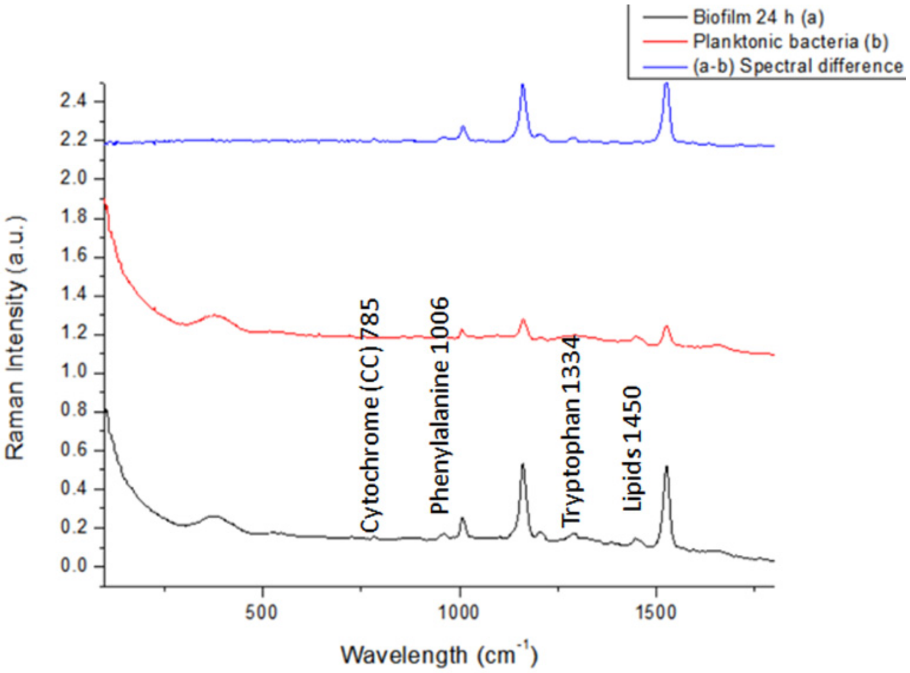

Supplementary Figure S4. Mean Raman spectra of MRSA in mature biofilm grown for 24 h (A) and planktonic bacterial cells incubated under the same conditions are compared. The spectral difference (A–B) is shown in (C)

To discriminate between the signals of biofilm cells from signals from planktonic cells, both spectra were analyzed by Raman spectroscopy. Interestingly, the signature peaks of the MRSA biofilm were marginal or absent at the beginning of biofilm growth (0–5 h). However, when the biofilm was grown for more than 4 hours, the signature peaks started to appear and helped to detect the formation of biofilm structure. In Fig. S4, the mean spectrum of MRSA biofilm grown for 24 h was compared with that of planktonic bacteria cells incubated in the same conditions. In Smith-Palmer et al., the comparison of cytochrome c (CC) to phenylalanine (Phe) from Raman spectra was used to determine the growth of biofilm on surfaces. The CC peak is distinctive at approximately 780 cm^{-1} and the Phe is a sharp signal at 1004 cm^{-1}. Biofilm EPS has previously been found to contain CC, as identified by Raman microscopy. To compare the proportions of EPS (CC) and protein (Phe) in a biofilm spectrum, the CC peak (747 cm^{-1}) and the Phe peak (1004 cm^{-1}) can be recorded. Further, the biofilm spectrum shows an intense peak at 1004 cm^{-1} associated with the ring breathing vibration of phenylalanine. Lastly, biofilm spectra differ significantly from those of planktonic cells, with signals related to a higher density of lipids at 1450 and 1334 cm^{-1}.

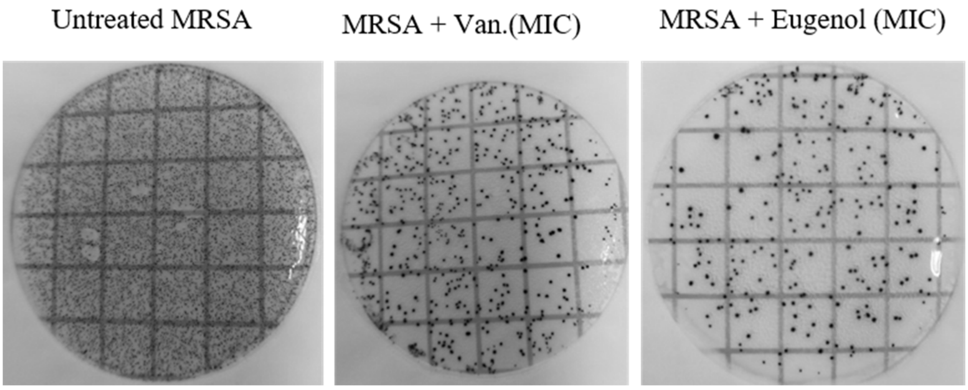

Supplementary Figure S5. Antibiofilm activity against MRSA biofilm on petrifilm (CFU/mL)

2 Supplementary Table

Supplementary Table S1. The cross-validation analysis of variance (CV-ANOVA) representing the cross validation result with p-value = 0

M1	SS	DF	MS	F	p	SD
**Total corr.**	334	334	1			1
**Regression**	307.89	32	9.62155	111.286	0	3.10186
**Residual**	226.1103	302	0.086458			0.294037

Reference

Smith-Palmer, T.; Lin, S.; Oguejiofor, I.; Leng, T.; Pustam, A.; Yang, J.; Graham, L. L.; Wyeth, R. C.; Bishop, C. D.; DeMont, M. E.; Pink, D.; In Situ Confocal Raman Microscopy of Hydrated Early Stages of Bacterial Biofilm Formation on Various Surfaces in a Flow Cell. *Appl. Spectrosc.* ***2016***, *70(2)*, 289–301. DOI: 10.1177/0003702815620539
